# Transactions of the Association of Fellows and Licentiates of the King's and Queen's College of Physicians in Ireland

**Published:** 1821-09-01

**Authors:** 


*Transactions of the Association of Fellows and Licentiates of the King's and Queen's College of Physicians in Ireland.* Vol. III. pp. 520. Dublin and London, 1820.

Our brethren, in Dublin, have lately produced the most authentic proofs of the zeal and talent with which medicine, in its comprehensive sense, is cultivated in the sister isle. Their writings have established the reputation of individuals, enhanced the fame of the Irish university, and enriched the archives of science. Acting upon equal talent, the discipline of the Dublin school of medicine must very soon produce effects which will be felt throughout the United Kingdom, and render the Irish academic qualification a badge of honour inferior to *none*—superior to most. Let other universities look to this. Talent is not hereditary—fame cannot be transmitted from father to son; nor can it be conferred by a cabal, or retained in name when lost in substance. We are arrived at that period, when intellectual, is nearly as ardent in operation, as mechanical competition. And as native capacity is equal, or nearly so, in great masses of society, so the institutions which give fullest scope to talent, impose least restraint on labour, and offer the surest reward for merit, must as regularly rise pre-eminent among others, as it is certain that plodding industry shall outstrip indolence, or genius dulness. Self-evident as these positions are in theory, and verified as they have often been by experience, they are *not* always put in practice. A spell seems sometimes to hang over a public body, as well as over a private individual, urging each to act contrary to the dictates of sober judgment.

> ———Video melioraque probo,
> Deteriora sequor.

Art. I. *Pathology and Treatment of Fever, founded principally on Observations, &c. By Robert Reid, M. D.*

We have dedicated so much space, in this Journal, to a delineation of writings on fever, that we must now be on our guard against extending this department beyond its proper bounds. At the same time we are unwilling to pass over any respectable monograph on this interesting subject, without giving the author that advantage which results from an extensive circulation of his more prominent doctrines and observations. Our circumscribed limits will therefore compel us to notice *only* the more striking traits of the paper now before us.

Dr. Reid, like the other Irish physicians who had ample experience, corroborates the fact of the late epidemic being decidedly contagious. Among the anomalies which it presented, were several cases of eruptive diseases, many of them so exactly resembling small-pox, that their identity would have been pronounced by any practitioner, if seen *only* at the period of maturation. In general, these patients did well, and required little or no medicine.* In some *few instances*, the eruption took on the appearance of pemphigus gangrenosus, and *these* cases proved mortal. In many instances, swellings in the neighbourhood of glands came on, and when to one of these were applied cold lotions, the febrile symptoms were exasperated in proportion as the heat, pain, and size of the tumour were lessened. This, of course, led to a change of treatment: leeches were applied instead of cold, "which soon effected some alleviation of the general symptoms." These tumours did not seem to be connected with any peculiar malignity in the disease.

Upon the *nature* of fever, that piece of knowledge "which still so near us, yet beyond us lies," our author has entered pretty fully, and in those inferences which appear deducible from the symptoms during life and the examinations after death, we agree very generally with Dr. Reid. But he goes a little farther into hypothesis than we can follow him, and then we consequently wish each other good bye. Considering the variety of type and character which fever assumes, under different circumstances, our author is led to view it, "as the result of a general cause, (he might have said a *variety of causes*,) which may be directed by accidental circumstances against some particular apparatus or function of the human frame."

"By considering fever to be strictly '*sine morbo locali primario*,' the endless disputes which have hitherto prevailed relative to the seat of fever, may, in some measure, be reconciled. An accurate investigation of the circumstances of the patient previous to the developement of fever and all the subsequent phenomena of the disease, tend to show some cause acting directly on the nerves, destroying their influence throughout the animal frame. The extreme vessels in the various organs are thus rendered incapable of resisting the force of the heart and arteries. This gives rise to congestion very different from active inflammation, the place of which is determined by the accidental debility of the part, or previous constitution of the patient." 19.

---

* We were somewhat surprized to see the editor of the Medical Intelligencer, No. 18, make the whole of these cases terminate fatally.

Dr. R. divides the nervous apparatus into three great branches or systems, the cerebral, spinal, and ganglionic, balancing each other, and, by their reciprocal influence, regulating the various functions. Although the causes of fever may fall on one, more than on another of these systems; yet when it is considered that not a single point exists in the animal frame that is not under the reciprocal influence of each and all of those nervous systems, we may easily conceive how difficult it must be to attach the symptoms of disease to their proper class. This is only to be done by long and patient observation at the bed side, assisted by dissection.

Our author believes that, upon an attentive contemplation of the phenomena of fever, four remarkable changes will be observed in the course of the disease.

"The first is, the immediate effect of the cause of fever, whether externally applied, or generated within the body of the individual. This may continue for an hour, for some days—nay, even some weeks! To this succeeds primary reaction, indicated sometimes by acuteness of the intellectual faculties and external senses, not unfrequently amounting to pain. Restlessness soon follows, which often induces the patient to exert himself considerably beyond what his strength is capable of supporting. The presence of this stage becomes principally conspicuous in the functions of the sanguiferous apparatus; the pulse feels laboured, with a sensation of throbbing, often very distressing in the head; the whole external surface appears distended, accompanied with considerable increase of heat. At this time also, there is frequently a troublesome cough, generally without expectoration.

"Should the vital powers of the constitution at this period rally, a disposition to active inflammation arises: this quickly attacks some organ or apparatus, and thus changes the character of the disease. Hither the whole force of the morbid actions are (is) directed, and all the symptoms arise which are peculiar to acute inflammation of the affected part.

"But it more frequently occurs, particularly among the inhabitants of large cities or manufacturing towns, that the general vital powers of the nervous centers still remain oppressed. Under such circumstances, when this stage has continued for a period long or short, according to the constitution of the individual or mode of treatment that has been adopted, some weak part gives way and local congestion takes place. Before this occurs there generally appears an exacerbation of all the symptoms; this, however, quickly subsides. The skin becomes relaxed, and often profuse perspiration bursts forth; the patient feels wearied, languid, and inclined to sleep; the pulse becomes feeble, sometimes quicker, but more regular, the natural evacuations take place, and thus a crisis may be observed."

But death often takes place instead of this crisis, and then dissection generally discloses local congestion, but not inflammation, unless the constitution should unexpectedly rally, soon after which, pains begin to fly about—thirst increases—the tongue becomes brown and furred—the alvine evacuations are suppressed, or discharged dark and fetid—the abdomen becomes distended—delirium—coma—convulsions or absolute exhaustion closes the fourth stage. Here dissection discovers "the most extensive appearances of inflammatory action." Purulent or sanious matter is sometimes found in the spinal cavity, but most generally congestion—in other viscera and cavities those marks of inflammation which are peculiar to their structures.

After many ingenious observations on the physiology of the cerebral system in health, which we must necessarily pass over, Dr. Reid proceeds to the deviations distinguishable during the existence of fever. A striking one is the altered expression of countenance, which is often observable long before the patient is aware of the impending disease. The appetite fails—the intellectual powers become confused—there is morbid sensibility, or an opposite state, over the surface of the body, &c. &c. &c. But we cannot follow our author through a long train of phenomena which are sufficiently familiar to the practitioner.

Dr. R. truly remarks, that it not unfrequently happens that traces of inflammation cannot be detected in the brain, *post mortem*, though the indications of its existence were unequivocal during life.

Our author considers it as of the utmost importance to ascertain whether the spinal system be the principal seat of disease in fever.

"Debility in the moving powers of the animal frame, whether relating to locomotion or organic structure, is one of the most constant indications of spinal disease in fever; this is principally conspicuous in the weakness and tremour of the limbs. As the disease advances, and congestion takes place in the spinal canal, general tetanic spasm frequently occurs. Should the disease at this stage prove fatal, upon examining the spinal cavity after death, a vascular congestion will be discovered between the dura mater and the parietes of the canal, accompanied with a firmness of the medullary mass. Sometimes the pia mater exhibits a tissue of minute vessels, which become intensely red after a few minutes exposure to the atmosphere; it has often happened, that the lower extremities have been affected with tetanic spasms, while the upper extremities and muscles of deglutition were totally relaxed. This generally causes a difficulty of swallowing, extremely resembling the paroxysm of hydrophobia: and is particularly conspicuous when the patient attempts to swallow liquids. From the observations I have had an opportunity of making, I am led to think, that patients whose constitutions had been injured by repeated attacks of intermittents, were most liable to this affection in continued fever." 41.

On the influence of the spinal system over the voluntary and involuntary muscles, thoracic and abdominal organs—on the state of the pulse—on petechiæ—on the temperature in fever, Dr. Reid makes many interesting observations, which our limits prevent being noticed; but before we speak of therapeutics, we shall make room for one more pathological extract, containing some curious matter.

"During the epidemic, which is the immediate subject of these pages, the liver seemed to be that organ of the ganglionic system which attracted the most constant attention of the physician. The actions of this viscus appeared to be modified according as the disease was severe upon the other systems. When the cerebral system was principally affected during the disease, the liver was found after death contracted and firm in its structure; the gall bladder contained but little fluid; this was sometimes a dark coloured, pitchy substance, and did not communicate the yellow stain to the neighbouring parts after death, which is usually observed when the gall bladder is filled with the proper biliary secretion. In these cases the blood did not appear deficient in quantity, and when drawn during life, had a tendency to become rapidly coagulated.

"When the spinal system was much affected, there was observed an excessive formation of biliary secretion, while the quantity of red blood appeared to be remarkably diminished. In such cases I have found in the gall bladder a number of small spicular stones, in appearance perfectly black: on minute examination, however, these were found to consist entirely of inspissated bile, about a grain of which was capable of giving a fine yellow colour to three quarts of water. The fluid in the gall bladder was thick, and nearly resembled black oil paint; but on being diluted with a large quantity of water, the whole became of a beautiful yellow colour. It may be extremely important for the practitioner to notice this circumstance, as in such cases the fæces may become apparently of the natural colour, although the biliary apparatus may not be in the slightest degree unloaded. The two first cases which occurred to me of this kind happened in the summer; both proved fatal. On examination after death, the liver seemed gorged with bile, and stellated; the sanguiferous vessels contained a considerable portion of biliary matter; the quantity of blood contained in the vessels throughout the body was so small and so changed in its sensible qualities, as to appear scarcely capable of performing any useful function in the animal economy; an attempt was made to take blood from the arms of both these patients, but without success. Such modification in the action of the liver indicates defective influence of the spinal system over that viscus. This seems well to coincide with the fact, that in comparative anatomy, as we descend in the scale of animated beings, it is found that the formation of red blood ceases to occur when the animals cease to be endowed with a vertebral column or spinal system." 60.

During the late epidemic, it was remarked that, in the remote parts of Ireland, where the poor were nearly destitute of medical assistance, and had little other sustenance than water, "the disease was seldom fatal." In cities, and among the upper classes, the fever was much more fatal, with all the assistance of medical art. This does not militate against the means used; but it proves that, in the *latter* classes of society, the constitution was less suited to bear the febrile movements with impunity, than in the *former* class. We have no doubt that if the upper classes were left destitute of medical means (we mean judicious means) the ratio of mortality would be greater. When any organ in them becomes inflamed or congested, Nature is not so equal to its preservation as in the lower classes, where less excitement is habitually applied to the system in general.

It is rare, of course, for the physician to be consulted in the very earliest movements of fever, or rather when its causes are operating in bringing forth the ostensible form of the disease. At such a period our author thinks that by serenity of mind, change of scene, and regulation of the bowels, the attack might be entirely repelled. When fever is actually developed, "absolute rest should be enjoined." In the first stage, the timely administration of an emetic often rouses the torpid energies of the frame, and recals the organs to energetic action, besides unloading the stomach of irritating sordes. "Should bleeding be resorted to in the period of collapse, a protracted disease must be inevitable."

"In fever, the first time that blood-letting from the general circulation can be used without doing mischief, is just previously to the second stage, when the pulse recovers energy, and there are symptoms of general reaction. Troublesome cough, without expectoration, is at this time often observed, generally attended with sharp pain about the chest. By taking twelve or fourteen ounces of blood from the arm, the patient will feel almost immediately relieved, and by supplying him with diluents which promote a gentle diaphoresis, at the same time regulating the operations of the alimentary canal, all the symptoms are mitigated, and the disease quietly proceeds to a termination without advancing to the congestive, or third stage." 65.

The third stage, in which local congestions in one or more viscera are prone to occur, is that which tries most the sagacity and resolution of the medical attendants. Congestion existing, but anterior to secondary reaction, general bleeding, Dr. R. thinks, is highly improper; local bleeding, however, is of the greatest advantage. In cerebral congestions, Dr. R. recommends the usual means; but considers blisters as highly prejudicial. "If the disease be severe, blisters to the head will not rise; when this happens they inevitably augment the morbid state they were intended to relieve, and very often induce a fatal apoplexy." When the force of the fever was exclusively directed against the cerebral system, a papular eruption on the back, breast, and loins, generally proved favourable. Where the spinal system was much oppressed, with torpor of the cerebral system, blisters were highly advantageous. Petechiæ our author looks upon as symptomatic of spinal debility; and as he considers the pelvic viscera to hold the most intimate connexion with the ganglionic, so he accounts for the utility of turpentine in such cases. He has seen patients, whose powers of life were so exhausted by disease, that deglutition was impracticable, gradually recalled to energy by purgative enemata with spirits of turpentine. In the treatment of the third stage of the disease, when the ganglionic system was chiefly effected, our author makes the following observations:—

"Soon, however, as pain in any part arises, or that it is excited by pressure, the stage of secondary reaction is to be expected: local bleeding by means of leeches now proves highly efficacious. I have had reason to imagine that the effect of leeches was more powerful than could be accounted for by the quantity of blood alone which they drew from the patient: this has been observed by Doctor Armstrong, and I have no reason to differ from his opinion. There are cases of this secondary reaction, where the process of sanguification appears to be arrested, and no blood can be obtained by opening a vein in the arm: even some cases which proved fatal, upon examination after death did not afford three ounces of proper red blood in the entire frame; yet these patients derived considerable relief during life from the application of leeches to the region of the liver. The internal medicines which appeared most efficacious in such cases, were calomel and opium combined, or a solution of tartarized antimony largely diluted. In the cases which terminated fatally while under the influence of these medicines, the internal surface of the stomach and alimentary canal exhibited a tissue of minute red vessels. It was extraordinary that this appearance extended from the stomach downwards in proportion to the length of time that the patient had been previously using these remedies. It may not be improbable that the French writers of the present day, who suppose that fever is seated in the mucous membrane of the alimentary canal, have been deceived by the appearance of vascular determination, which the remedies they employed during the disease induced upon the internal surface of that canal." 82.

Here our analysis must close. The remainder of the paper is occupied with an investigation of the means of arresting the progress of contagious fever, when epidemic in a country; and for this investigation we must refer to the volume itself. We have adduced enough to shew that Dr. Reid is no common observer, and that about him there is no lack of either zeal or talent. We are not quite certain that the ingenious author, like all other ingenious writers, has not a little bias or bend towards a favourite theory. We will not pretend to determine whether the bend be sinister or dexter, in his medical escutcheon—or whether it may not incline a little *spinally;* but we may avow our belief, that it has not had any influence in causing him to deviate from the sober path of sound judgment in the paramount object of all investigations—the successful treatment of disease.

---

Art. II. *Case of Hæmorrhage, supposed to be from the Spleen.* By W. Harrison, M.D.

As this case does not appear to us to involve any particular practical question, we shall be very concise in our notice of it. A sergeant of dragoons, accustomed to hard riding, had been dragged off his seat; fell with violence to the ground; and received a hurt in the region of the spleen, after which, for ten or twelve days, he had felt pain and soreness, with a sense of scalding, at the scrobiculus cordis. At the expiration of this period, he was seized with a discharge of blood, to a great amount, upwards and downwards. When seen by Dr. H. the pulse was scarcely perceptible—face and lips exsanguious—voice sunk—articulation unintelligible. He rallied a little from this state; but next day had a return of the hæmorrhage. On the third day, febrile symptoms set in, with pain and tenderness in the splenic region, and tension of the abdomen. Blood was abstracted from the arm, *ad deliquium animi;* and from that time he gradually convalesced.

We do not think there is any thing in the case to found more than a mere conjecture on, that the hæmorrhage proceeded from the spleen. We have seen such vast quantities of blood issue, in a short time, from the mucous membrane of the primæ viæ, in severe dysenteric affections, that we can find no difficulty in believing the hæmorrhage, in the present case, to have emanated from that source.

---

ART. III. *Cases of Eruptive Diseases.* By DR. ROBINSON.

These cases are four; two of variolous and two of varioloid eruption. The *latter* were post vaccinam. The distinctions between the *two species* were so remarkable, that the latter, he thinks, may be considered a *new*, or mild species of the disease. These, with similar facts furnished by Dr. Hennen and others, lead to the following conclusions:—

"1. That an eruptive disease is produced by the contagion of small-pox on the persons of *some*, who had undergone the vaccine inoculation.

"2. That this eruption has, in a certain degree, the form, and effects the course and changes of variola, although of *shorter duration*, but is not *dangerous* to human life, and may be considered a *new** and mild species of that disease.

"3. That vaccination, as far as our experience has yet gone, appears fully capable of preventing the *fatal effects* of small-pox, and of ultimately banishing that formidable and loathsome disease from among mankind." 111.

Dr. Robinson inoculated thirty children belonging to the same school, (eighteen of whom had been vaccinated several years previously, the rest had had small-pox,) with variolous matter. The result was the same in all—no eruptive disease was produced, nor affection, local or constitutional. "This experiment, he observes, is decisive in favour of vaccination."

ART. IV. *A Case of Emphysema, without external violence.* By RICHARD STANLEY IRELAND, M.D.

Cases of this kind are comparatively rare. A child, nine years of age, was attacked with pneumonia, the violent inflammatory symptoms appearing to give way to the usual remedies, but a troublesome and distressing cough remaining, after a severe fit of which, a colourless swelling was observed above the clavicles. This swelling rapidly increased, extending up one side of the neck to the face, immediately after which, the entire scalp became emphysematous, rendered unequivocal by crepitation. The respiration now became more difficult, and the cough doubly distressing—the pulse quicker, smaller, indistinct, and irregular—the face pale, and ultimately livid, with cold perspirations over the

* Doctor Clarke calls it, a *Hybrid*.

body. The little patient was bled largely by leeches every day, with temporary benefit. Scarifications would not be permitted, and the child died on the fifth day. Examination of the body was not allowed.

Dr. Ireland conceives that the emphysema was produced by the rupture of an air vessel in the lungs, from the violent exertion of coughing; and that the air became diffused through the cellular substance connecting the lobules, insinuating itself upwards between the pleura pulmonalis and lungs, at the root of which it escaped, occupying the space between the two layers of the mediastinum, whence it appeared above the clavicles, and soon extended to the parts before-mentioned. Dr. Ireland remarks that he never before saw a case of this kind, nor does he recollect to have read of any. If he or the reader will turn to the 12th volume of the Dictionnaire des Sciences Medicales, page 6, *et seq.* he will find many curious cases and particulars illustrative of this subject. The following case, lately attended by the celebrated Dubois of Paris, is exactly in point.

"A child, thirty-two months old, was seized, in the beginning of July 1812, with violent convulsive cough, the paroxysms being of frequent recurrence. On the morning of the fifth day, a few moments after a violent paroxysm, emphysema appeared at the upper part of the sternum, whence it quickly extended under the nastoideus muscle to the face—to the arm pit—and all the upper parts of the chest. In the evening of the same day, the emphysema was found to occupy the neck, the abdominal parietes, and the scrotum. The following day the upper and lower extremities were emphysematous, and the difficulty of breathing increased with the progress of the emphysema. Suffocation became imminent, and tracheotomy was impracticable, from the great swelling of the neck; besides, such an operation appeared useless, as the ruptured vesicle was at a great distance below. Dubois therefore only recommended anodyne and pectoral drinks to assuage the cough, and the whole body to be enveloped in compresses soaked in aromatic wine. Under this treatment the respiration became gradually more free, and in the space of eight days the emphysema totally disappeared." *Op. Cit. p.* 8.

Bromfield, Benjamin Bell, and some other authors, have stated that these accidents result occasionally from violent fits of coughing, crying, or even immoderate laughter—also in consequence of obstruction in the trachea. Hoffman relates a case of rupture of an air-cell and suffocation as the termination. Meckel (Memoirs of the Royal Academy of Berlin, vol. vii. p. 16,) states a case of emphysema where, in consequence of an obstruction of mucus in the trachea, air burst into the cavity of the pleura of the right side, and suffocated the patient.

The fifth article in the volume before us, is on affections of the cranial brain, by Dr. Nicholl, which forms the basis of a small volume that will be found reviewed in another part of our Journal. We shall take up this third volume of transactions in our next number, and hope to finish the analysis of it in that article.

